# Extracellular Matrix Deposition in Engineered Micromass Cartilage Pellet Cultures: Measurements and Modelling

**DOI:** 10.1371/journal.pone.0147302

**Published:** 2016-02-18

**Authors:** Miranda C. Lewis, Ben D. MacArthur, Rahul S. Tare, Richard O. C. Oreffo, Colin P. Please

**Affiliations:** 1 Mathematical Sciences, University of Southampton, Southampton, SO17 1BJ, United Kingdom; 2 Centre for Human Development, Stem Cells and Regeneration, Faculty of Medicine, University of Southampton, Southampton, SO16 6YD, United Kingdom; 3 Mathematical Institute, University of Oxford, Oxford, OX2 6GG, United Kingdom; University of Crete, GREECE

## Abstract

This article explores possible mechanisms governing extracellular matrix deposition in engineered cartilaginous cell pellets. A theoretical investigation is carried out alongside an experimental study measuring proteoglycan and collagen volume fractions within murine chondrogenic (ATDC-5) cell pellets. The simple mathematical model, which adopts a nutrient-dependent proteoglycan production rate, successfully reproduces the periphery-dominated proteoglycan deposition, characteristic of the growth pattern observed experimentally within pellets after 21 days of culture. The results suggest that this inhomogeneous proteoglycan production is due to nutrient deficiencies at the pellet centre. Our model analysis further indicates that a spatially uniform distribution of proteoglycan matrix could be maintained by initiating the culture process with a smaller-sized pellet. Finally, possible extensions are put forward with an aim to improve the model predictions for the early behaviour, where different mechanisms appear to dominate the matrix production within the pellets.

## Introduction

In recent years, much effort has been directed towards overcoming the clinical challenges associated with articular cartilage injury. Although current treatment protocols can provide temporary relief of symptoms, they are incapable of restoring long-term function to damaged cartilage. For this reason, and due to its relatively simple structure, of a single cell type in an extracellular matrix devoid of blood vessels, nerves or lymph vessels, articular cartilage is considered a promising candidate for tissue engineering.

The aim of cartilage tissue engineering is to construct functional tissue implants for long-lasting repair of full-thickness defects. For clinical reimplantation, the tissue-engineered (TE) constructs must possess the material properties matching those of native cartilage. In particular, the load-bearing function is dependent on the highly viscous and low compressibility properties of the extracellular matrix (ECM) surrounding the cells. The matrix is composed of collagens, proteoglycans and noncollagenous proteins, each of which differs in their mechanical properties and the roles they play within this three-dimensional network.

Several experimental studies have measured the concentration distributions of collagen and proteoglycans to identify mechanisms controlling cartilaginous matrix development in TE constructs. However, to date, many of the factors involved in regulating matrix synthesis, deposition, and degradation are either unknown or poorly understood [1].

Various studies have looked at the evolution of the different matrix components in TE constructs, employing mathematical models to gain insight into possible governing parameters. Obradovic *et al*. [1] used reaction-diffusion equations of oxygen and glycosaminoglycans (GAG) to explore oxygen-dependent deposition of GAG in polymer scaffolds seeded with bovine chondrocytes. The model predictions of GAG distributions were compared with experimental measurements and provided a good fit to the data, supporting the hypothesis of a first-order dependence of GAG synthesis on oxygen concentration. However, it should be noted that no comparison was made between the predicted oxygen profiles and corresponding experimental data. Furthermore, the model did not predict cellular dynamics, but rather used interpolated values of experimentally measured cell number densities. Although observed cell density profiles and the considerable increase in construct volume indicated that cell movement played a crucial role in the dynamics of the tissue development, the model did not take this parameter into account.

Wilson *et al*. formulated mathematical models to describe matrix accumulation and scaffold degradation in cartilage cell-polymer constructs [2]. Motivated by experimental evidence [3,4], the ECM deposition model included the effects of product inhibition, characterised by a negative correlation between GAG molecule deposition and its synthesis rate. The model successfully predicted temporal changes of the total construct mass, but neglected spatial variations in tissue composition. Cell proliferation and death were not considered, neither were interactions between the chondrocytes and the matrix molecules.

Similarly, Saha *et al*. observed [5,6] the important role of ECM in regulating chondrocyte proliferation, differentiation and homeostasis [7] and constructed a simple model based on a negative feedback control mechanism, to study cell-matrix interactions [5]. Subsequently, the effects of growth factors on biomolecule production were also included [6]. Given the limited knowledge about the functional characteristics of these growth factors, their effect was introduced as random fluctuations in the form of Gaussian white noise. The results showed that, in the studied regime, the growth factors inhibited the steady state production of collagen and GAG molecules, in accord with experimental data. The model was subsequently extended to apply the ‘growth factor effect’ only to the growth rates of the ECM molecules as distinguished from a decay rate [8]. These studies from Saha *et al* set out to establish a predictive tool for parametrically comparing experimental results and helping assess the culture times necessary to produce a functionally viable construct. However, due to the large number of parameters required in the models, this could only provide limited insight into characterising the possible mechanisms; their models ignore the spatial composition of the tissue, cellular dynamics and the diffusion-consumption of an external nutrient.

Pisu *et al*. [9] extended the model by Obradovic *et al*. [1], formulating material balances for the oxygen and GAG concentrations, coupled with mass structured population balances. Cellular proliferation and death were included to evaluate cell density profiles within the scaffold. Cell movement, however, was neglected, and spatial growth was taken into account by locally evaluating the volume occupied by the tissue. The model did not indicate how environmental changes affect the nutrient transport and growth processes within the tissue. The comparison between model predictions and experimental data was restricted to the case where limited growth had taken place. Pisu and colleagues [10] also developed an extended model which accounts for collagen production and produced simulations that compare with experimental data in various static culture systems. This study demonstrated that their general modelling approach could also be applied to different scaffolds and system configurations.

The work presented in this article investigates the spatial and temporal evolution of extracellular matrix within cartilaginous cell pellets. A simple mathematical model is formulated, following a similar approach to Obradovic *et al*. [1] and Pisu *et al*. [9], to identify key parameters governing tissue development within pellets of murine chondrogenic cells (ATDC-5). However, unlike the models above, this study considers the dynamics associated with cellular motion in the growing tissue. In addition, we do not assume matrix production to depend expressly on *oxygen* concentration, but adopt a more general approach where the building block controlling matrix synthesis remains unspecified. The model formulation was performed alongside the experimental programme described below to measure collagen and proteoglycan distributions within ATDC-5 pellets cultured for 28 days.

## Materials and Methods

### Pellet culture

Murine chondrogenic ATDC-5 cells were first expanded in monolayers, and then harvested for pellet culture. Cell pellets were created by centrifuging 8×10^5^ cells, and were cultured in chondrogenic medium for 28 days in a humidified incubator at 37°C and 5% CO_2_ as previously detailed [11]. After 2–3 days, the cells aggregated to form a compact mass of approximately 100 *μ*m in diameter. The pellet was regularly stirred within the medium to ensure nutrient access to all sides and to prevent it from adhering to the walls of the tube. Once a compact pellet had formed, the chondrogenic medium was renewed every 3 days over the 28-day period of culture. At each of days 7, 14, 21 and 28, three pellets were harvested for histological analysis.

### Histological Analysis

**Fig 1:**
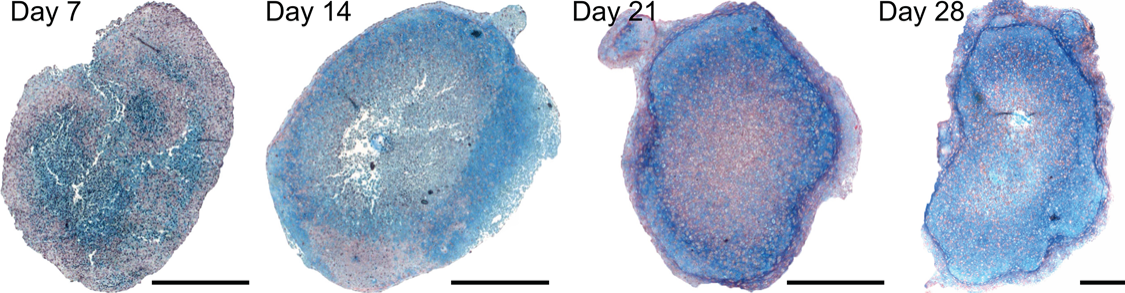
Time-course of chondrogenic differentiation of murine ATDC5 cells in pellet culture. Pellets, harvested at days 7, 14, 21 and 28 of culture, were stained with Alcian blue and Sirius red to demonstrate presence of proteoglycans and collagen, respectively, in the cartilaginous tissue. Scale bar: 100μm.

For histological evaluation, the pellets were fixed in 4% phosphate-buffered paraformaldehyde; and sections were cut at 5 *μ*m for staining. Alcian blue was used for staining the proteoglycan-rich matrix, and Sirius red for collagen staining. Samples were visualised and photographed using a Zeiss Axiovert 200 microscope. Fig 1 shows representative pellet staining at the different time-points.

### Image Analysis

Proteoglycan and collagen distributions in pellets were evaluated using high-resolution image analysis on the stained sections. Image analysis (IA) was performed to determine the spatial distribution of matrix elements by fitting a circle around the given sample and dividing it into a series of 5 to 6 concentric rings of equal radial width. The volume fractions of blue (proteoglycans) and red (collagen) were then measured in each of the annular rings. In addition, an assessment of the total area of the pellet section was obtained. Measurements were taken for the three pellet groups at days 7, 14, 21 and 28, to quantify the spatiotemporal evolution of collagen and cartilaginous matrix deposition within ATDC-5 cell pellets.

At each time-point between 4 and 14 representative sections through the centre of each replicate pellet were analysed, and the mean staining intensities for proteoglycans and collagen were recorded.

## Results

### Experimental results

**Fig 2:**
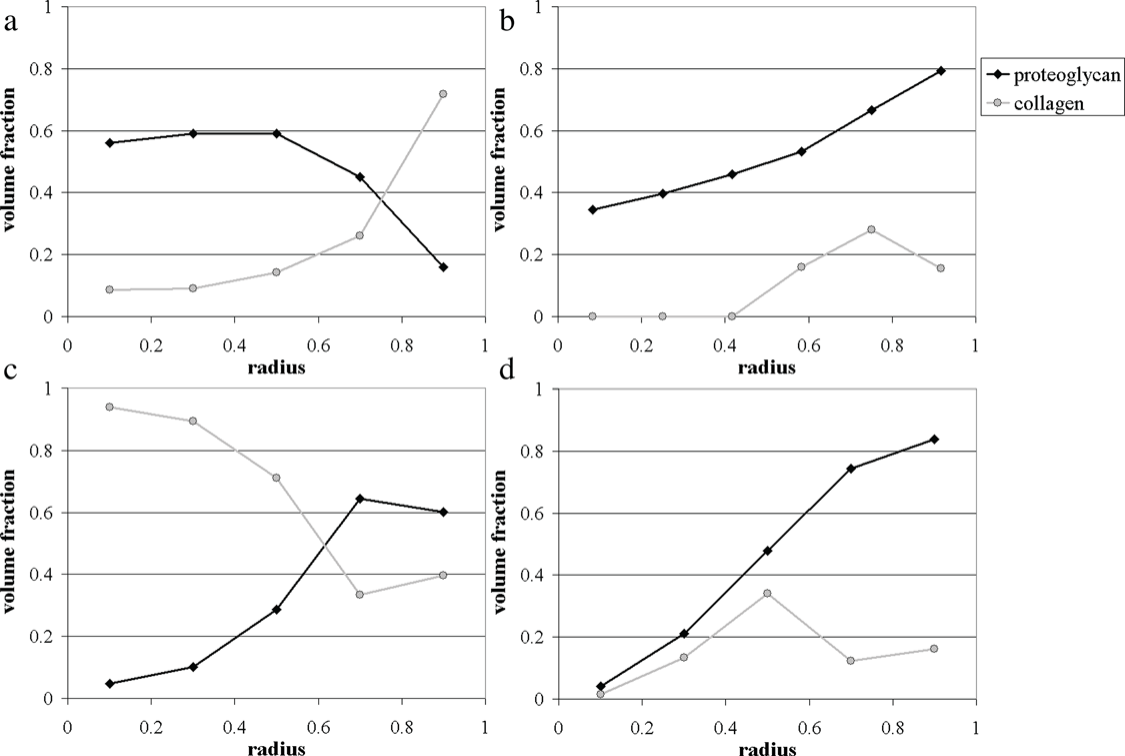
Experimental measurements of proteoglycan and collagen volume fractions within ATDC-5 pellets cultured for 7, 14, 21 and 28 days. In all panels the x-axis corresponds to the non-dimensional radius *r* / *S*(*t*). (a) Day 7; (b) Day 14; (c) Day 21; (d) Day 28.

At the initial harvesting of pellets, after 7 days in culture, pellet constructs were observed to be approximately spherical in shape, with an average diameter of ~ 150 *μ*m. Histological assessment of the stained sections demonstrated the presence of collagen and proteoglycans throughout the pellets. The image analysis demonstrated that, at this stage, a distinct structure in the matrix deposition was already beginning to emerge, whereby the peripheral layer of the pellets consisted mainly of collagenous tissue (Sirius red positive staining), whilst the centre was predominantly stained for proteoglycan-rich matrix (Alcian blue positive). The measurements presented in Fig 2(A) reveal a well-defined pattern of matrix deposition, where the four central subregions of the pellet are primarily made up of proteoglycan-rich matrix (40–75%), and the outer rim of the pellet is chiefly collagenous in nature, with the blue-stained proteoglycans occupying only ~ 15% of this region.

After 14 days of cultivation, the aggregates were observed to have increased in size, measuring on average ~ 220 *μ*m in diameter. Each sample consisted of a proteoglycan rich peripheral band of cartilaginous matrix surrounding a ring-shaped region, which displayed concomitant deposition of proteoglycans as well as collagen. The centres of the pellets were almost devoid of the stained matrix components. The unstained regions in the centre represented either a result of tearing occurring when pellets were sectioned, or an accumulation of necrotic matter. The data generated from the image analysis delineated a clear structure in the matrix layout (see Fig 2(B)). Proteoglycans were present throughout the pellet, decreasing progressively in volume fraction from the surface inwards (from 70–90% at the periphery of the pellet to 25–45% at the centre). The central region was completely devoid of collagen evidenced by negligible Sirius red staining, while collagen was measured in the peripheral regions (subdivisions 4, 5 and 6) in smaller quantities relative to the proteoglycans.

After 21 days in culture, pellets (200–250 *μ*m) displayed a central region of predominantly devoid of both matrix components, surrounded by a proteoglycan-rich matrix. A thin layer of fibrous collagenous tissue appeared to envelop the outer surface of the pellet. As observed in the 14 day samples, the centre of the pellets presented areas devoid of both matrix components, most likely corresponding to the emergence of a necrotic core. The results from the image analysis in Fig 2(C) outline the appearance of a boundary between subregions 3 and 4, demarking a central region dominated by collagen (60–100%), surrounded by an outer layer of cartilaginous matrix abundant in proteoglycans (~70%).

After 28 days in culture, the ATDC-5 pellets had grown further in size (~270 *μ*m), displaying a similar matrix layout to those harvested after 21 days of culture. The amount of proteoglycans deposited had significantly increased, occupying the majority of the peripheral region of the pellet (Fig 2(D)). Collagen was present predominantly in the middle ring (subsection 3); whilst collagen and proteoglycans were absent in the pellet centre, which appeared to be necrotic.

### Mathematical Modelling

The following describes a simple mathematical model that explores possible mechanisms to explain the experimental observations outlined above. Although little is known about the structural make-up and metabolic mechanisms of the ECM components within articular cartilage, the use of the accompanying experimental observations allows exploitation of a number of simplifying assumptions to our modelling. We note that collagen forms part of a tensile network of interconnected fibrils, whilst proteoglycan matrix resembles a highly hydrated gel-like material [12]. For the sake of simplicity, we will limit our modelling to the dynamics of a single matrix component, focussing on the evolution of the proteoglycan-rich matrix because of its viscous nature, rather than the collagen fibrous network that appeared immobile and virtually unchanged over the course of culture. We shall not consider the complications associated with cellular differentiation, but assume all cells have differentiated and become specialised producers of ECM. It is useful to note that our model can be viewed more generally as a description of the dynamics between cells and the extracellular matrix they produce within a growing tissue. For simplicity, we describe these dynamics by considering the tissue to be made of just two elements quantified by volume fractions, namely the cells, denoted by *n*, and the proteoglycan matrix, labelled as *p*. We recognise this is a gross approximation to the tissue composition, but we believe that taking a very simple modelling approach will enable us to gain more insight into the key mechanisms that control the tissue development.

In the experiments detailed above, micromass pellets were typically spherical in shape, thus allowing us to assume spherical symmetry and to use radial coordinates (radius *r* and time *t*) to formulate the model equations. To account for cellular dynamics within the tissue, a number of assumptions must be made, due to the absence of relevant experimental parameters. In the existing literature, various studies using chondrocytes and mesenchymal progenitor cells have reported that cellular proliferation within TE constructs occurred predominantly during the first 5 days of culture, after which the cells gradually ceased proliferating and commenced to differentiate, represented by the synthesis of extracellular matrix components [1, 13–15]. In light of this, and given that we are considering pellets harvested after 7 days of culture, we have decided to remove cell proliferation and death in the model description. This approximation is further supported by a general examination of the stained samples, from days 7 and 14, showing no dramatic increase in the cell-population size.

With regard to the metabolic mechanisms, several studies have established a marked correlation between matrix production and energy metabolism [16]. In particular, proteoglycan production is thought to depend on nutrition [17], pH [18], and oxygen [1, 19–20]. Unfortunately, accurate quantifications of the uptake and production of basic metabolites such as oxygen, glucose and lactate are currently lacking in the literature. In their modelling work, Obradovic *et al*. [1] formulated local GAG kinetics as product-inhibited, with a Michaelis-Menten dependence on oxygen concentration. In the present investigation, we do not attribute matrix production to a specific metabolite, but consider a generic local building block, whose concentration is denoted by *B*(*r*, *t*). This variable, *B*(*r*, *t*), represents the critical nutrient that controls proteoglycan matrix synthesis. It could denote oxygen, glucose, or any other key constituent put forward in the literature. Such a simplification does not significantly influence the predictive capacity of the model and accommodates for the lack of experimental evidence on the parameters governing matrix production.

The nutrient *B*(*r*, *t*) is assumed to move due to diffusion, with a constant diffusion coefficient *D*, and to be consumed by the cells at a rate *Q*. We assume that nutrient consumption is the dominant mechanism for matrix synthesis, such that the cells produce proteoglycan matrix at a rate *P* proportional to *Q*. Following the usual approach for describing oxygen and glucose consumption rates in tissue [1, 16, 21], we apply Michaelis-Menten kinetics and define *P* and *Q* as

$$Q=\frac{\alpha B}{\kappa_{0}+B}H(B)n,\;P=\frac{\beta B}{\kappa_{0}+B}H(B)n$$

where *α*, *β* and *κ*_0_ are constants. The function *H*(.) is the Heaviside function with (*H*(*B*) = 1 if *B* > 0, *H*(*B*) = 0 if *B* ≤ 0) and indicates that matrix cannot degrade to create the nutrient.

We make the simplifying assumption that cells and proteoglycan matrix move at the same velocity $\underline{u}=(v(r,t),0,0)$. Initially, the pellet is taken to be composed of cells exclusively, with no matrix present, and to have radius *S*(*t*) = *S*_0_. We impose no flux of nutrient and zero velocity at the centre of the pellet to account for its symmetry. Since the culture medium surrounding the pellet is regularly stirred, the nutrient concentration, *B*(*r*, *t*), is assumed to be continuous across the boundary of the pellet and equal to the concentration in the surrounding medium *B*_0_. Finally, we apply the kinematic condition at the pellet surface, such that the outer boundary, *S*(*t*), is taken to move at the velocity of the cells of which it is composed.

The full model is outlined in the supporting information (S1 Text), and suitable scalings are defined. The subsequent nondimensional system, which is used to produce the numerical simulations, is given by

$$\xi \left[\frac{\partial \tilde{B}}{\partial \tilde{t}}+\nabla \cdot (\underline{\tilde{u}}\tilde{B})\right]=\nabla^{2}\tilde{B}-\frac{\tilde{B}}{\kappa +\tilde{B}}H(\tilde{B})\tilde{n}$$ (1)

$$\frac{\partial \tilde{n}}{\partial \tilde{t}}+\nabla \cdot (\underline{\tilde{u}}\;\tilde{n})=0$$ (2)

$$\nabla \cdot \underline{\tilde{u}}=\frac{\tilde{B}}{\kappa +\tilde{B}}H(\tilde{B})\tilde{n}$$ (3)

with $\tilde{B}=1$ and $\tilde{v}=\frac{d\tilde{S}}{d\tilde{t}}$ at $\tilde{r}=\tilde{S}$, $\tilde{v}=0$ and $\frac{\partial \tilde{B}}{\partial \tilde{r}}=0$ at $\tilde{r}=0$, and $\tilde{S}=\Sigma_{0}$, $\tilde{n}=1$ and $\tilde{p}=0$ at $\tilde{t}=0$ where

$$\xi =\frac{\beta B_{0}}{\alpha }\;,\;\kappa =\frac{\kappa_{0}}{B_{0}}\;\text{and}\;\Sigma_{0}=\frac{S_{0}}{L}\;,$$

and tildes denote dimensionless quantities. The parameter *ξ* measures the rate of matrix production relative to the nutrient consumption rate. There are currently no available data for this parameter. However, based on intuition, and in order to simplify the model analysis, we assume that nutrient consumption occurs at a much faster rate than matrix production, thereby neglecting the time derivative and advective term in Eq 1 by taking *ξ* = 0. This could be interpreted as assuming that cells consume a lot of nutrient in order to synthesise just a small amount of proteoglycan-rich matrix. It is assumed that as cells differentiate they become specialised producers of ECM in comparison to progenitor cells and will have different nutrient needs.

Therefore, there are only two parameters left in the model, namely *κ* and Σ_0_. The value of *κ* determines how fast the nutrient consumption rate and the matrix production rate drop as the nutrient concentration gets close to zero. The parameter Σ_0_ corresponds to the ratio of the initial pellet radius with the nutrient diffusion length scale. Varying this parameter allows us to study the effect of the initial pellet size on the spatiotemporal evolution of the matrix distribution within the pellet.

### Model Comparison with Experimental Results

The simple mathematical model proposed in this paper, was formulated to help interpret the proteoglycan patterns that developed within ATDC-5 cell pellets. The model was solved numerically, by applying a simple finite-difference scheme with implicit time stepping.

We found that the model fits well the matrix layout observed after 21 and 28 days, but the predicted early behaviour does not concur with experimental measurements for days 7 and 14. Fitting the model to the experimental results has therefore required some interpretation.

Given that our model reproduces only the behaviour observed on days 21 and 28, we used the experimental data from these two timepoints to fit the theoretical predictions. The data fitting was performed informally, by prescribing the values of four parameters, namely, the initial pellet radius *S*_0_, the proteoglycan production rate constant *β*, the nutrient diffusion length scale *L* and the parameter *κ* which determines the slope of the Michaelis-Menten function. Fig 2(A) and 2(B) show the experimental and theoretical results for the proteoglycan matrix profiles at days 7, 14, 21 and 28, mapped on a fixed [0,1] radius (scaled with the radius of the pellet at each timepoint). The parameter values used in the model were:

$$S_{0}=84\;\mu \text{m},\;\beta =0.23\;{\text{s}}^{-1},\;L=14\;\mu \text{m and}\;\kappa =0.3.$$

**Fig 3:**
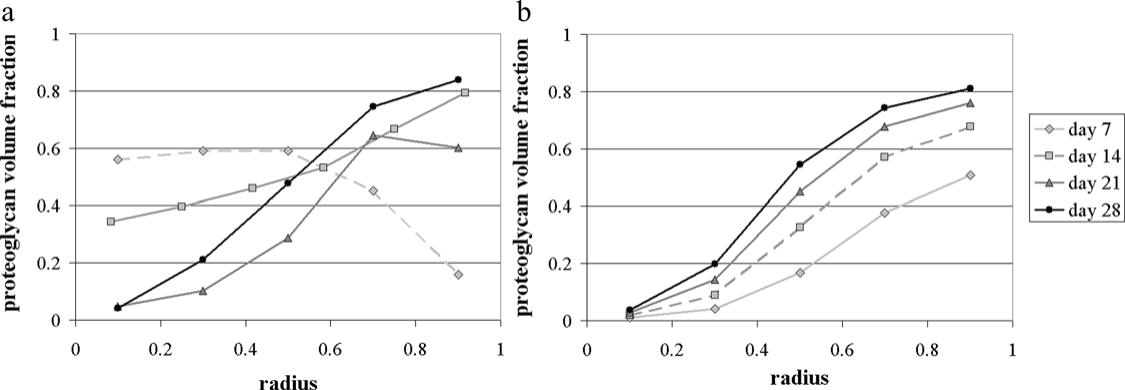
Proteoglycan volume fraction within pellets cultured for 7, 14, 21 and 28 days: comparison between the experimental data (mean volume fraction over three replicates) (a) and our model predictions (b), with Σ_0_ = 6, *β* = 0.23 s^-1^ and *κ* = 0.3. In both panels the x-axis corresponds to the non-dimensional radius *r* / *S*(*t*). (a) Experimental data; (b) Model predictions.

**Fig 4:**
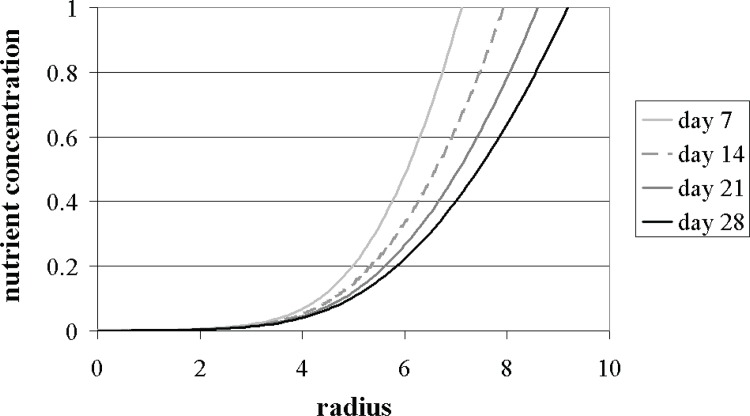
Numerical predictions for the nutrient concentration profiles within a growing cell-pellet after 7, 14, 21 and 28 days of culture, with Σ_0_ = 6, *β* = 0.23 s^-1^ and *κ* = 0.3. The nondimensional radius corresponds to *r* / *S*(*t*).

As can be seen in Fig 3, our simple model successfully predicts the general trend of the behaviour observed during the later culture period (after 21 days), characterised by the outward deposition of proteoglycan matrix at the pellet periphery. The predicted nutrient concentration distributions are also presented in Fig 4. Unfortunately, no experimental measurements were available to allow a comparison with these results. The theoretical results for days 7 and 14 require some additional consideration.

At day 7, the mathematical model predicts that the amount of proteoglycan matrix gradually increases from the centre of the pellet outwards (see Fig 3(B)). The experimental results, however, indicate that after 7 days the pellets presented a concomitant production of collagen and proteoglycans, with more collagen synthesised near the construct periphery. We believe this could be related to the fact that the pellets are initially formed with cells in a predominantly proliferative state maintained for optimal cell growth in basal medium, which subsequently switch to a predominantly metabolic (active differentiation) phenotype. Thus, in order to produce proteoglycan matrix, the cells need to switch to active differentiation. It is likely, however, that this “active differentiation” does not occur in a uniform manner, but that it relies on cell-cell signalling to spread to different regions. In this case, the irregular patterns of proteoglycan matrix observed at day 7 would be a consequence of that activation, rather than of the mechanisms included in our model. By day 14, the measured proteoglycan profile suggests that the nutrient-dependence of matrix synthesis, considered in the model, begins to influence the matrix deposition, localising it to the peripheral layer of the pellet, where nutrient concentrations are highest (see Fig 3). This behaviour was further accentuated by day 21. The periphery-dominated matrix production, characteristic of the proteoglycan build-up in pellets after 21 and 28 days of culture, was successfully predicted by the model as demonstrated in Fig 3(B).

The slight reduction in the volume fraction of proteoglycan matrix measured in the outer band of the day 21 pellets corresponds to the presence of a thin layer of fibrous collagenous tissue enveloping the outer surface of the pellet. Several investigations using cell clusters have reported morphological changes in the surface cells, characterised by an elongated fibroblast-like shape [22, 23]. This altered cellular differentiation profile may explain the production of a different matrix component (collagen) at the pellet surface. In this study, however, cellular differentiation processes were not included.

## Discussion and Conclusions

Our model predictions reveal a number of interesting growth dynamics occurring within cell pellets during *in vitro* culture. The model assumes that the nutrient consumption and proteoglycan matrix production rates have the same dependency on the nutrient concentration. The parameter regime that fits the experimental data corresponds to the case where the initial pellet size is large enough to produce sharp gradients in nutrient concentration right from the start of culture. Although the precise mechanisms have yet to be determined, these results suggest that while the peripheral region of the pellet is supplied with sufficient nutrient to yield extensive proteoglycan matrix production, the shortage of nutrient at the centre, caused by diffusional limitations, produces a central core where virtually no proteoglycan matrix is synthesised.

Numerical simulations showed that the general behaviour of the model is essentially unaffected by variations in the parameter *κ*. The model was analysed, keeping *κ* fixed, and varying Σ_0_, to study the effect of the initial pellet size on the evolving matrix and nutrient distributions. An interesting finding is the model prediction that periphery-dominated matrix deposition could be avoided by initiating the culture process with a smaller-sized pellet.

**Fig 5:**
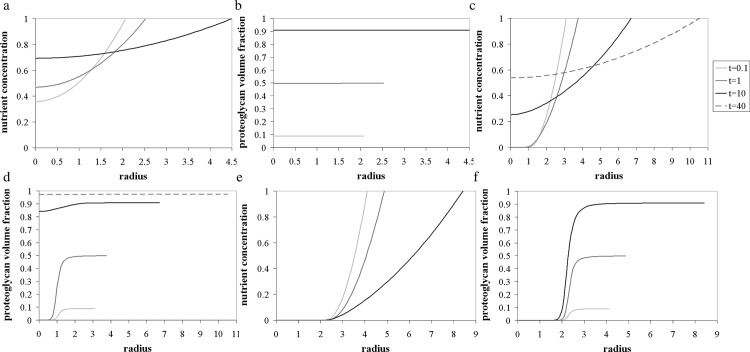
Model results describing the evolving distributions of the nutrient concentration (a, c and e) and the proteoglycan volume fraction (b, d and f) within pellets for different initial aggregate radii, with *κ* = 0.005 and Σ_0_ = 2, 3 and 4. (a-b) Σ_0_ = 2; (c-d) Σ_0_ = 3; (e-f) Σ_0_ = 4.

For example, if the initial pellet radius is taken to be half that of the ATDC-5 pellets considered here, the interior is still deprived of nutrients at the start of culture, causing the cells to produce more matrix near the surface, where nutrient is plentiful. As time evolves, however, and matrix is synthesised at the periphery, the local cell volume fraction in that region decreases, as does the amount of nutrient consumed, yielding a gradual rise of the nutrient concentration profile (see Fig 5(C)). The increase in the amount of nutrient reaching the centre activates the cells in this region to produce proteoglycan matrix, at a rate proportional to their volume fraction. This dynamic dependence of matrix production on cell volume fraction and nutrient concentration therefore allows the initial nutrient limitations to be gradually overcome. The matrix distribution then becomes almost uniform across the pellet, if it is cultured over a long period of time (Fig 5(D)). However, if the culture process is initiated with a larger sized pellet, the centre cannot be replenished with nutrient, due to the width of the nutrient depleted region at the start of culture. In such cases, the matrix production is confined to the outer ring throughout the culture period (see Fig 5(F)). On the other hand, if the pellet were formed with an initial radius small enough to prevent any nutrient deficiencies at the centre from the start of culture, the numerical solutions predict that an almost uniform distribution of proteoglycan matrix could be maintained throughout the cultivation period (see Fig 5(B)). This crucial result suggests that larger pellets of uniform matrix layout could be achieved by cultivating several smaller pellets, and conglomerating them once their local cell volume fraction is small enough to prevent the emergence of nutrient gradients.

Taken together, our model analysis indicates additional mechanisms are likely to control the synthesis of extracellular matrix components during the early stages of culture. In particular, cellular activation to a fully active differentiated phenotype, and the corresponding production of the different matrix components, could represent a promising avenue to address the limitations of our current model. The factors that trigger cellular redifferentiation are yet to be uncovered; although cell-cell contact and the secretion of growth factors are likely to play crucial roles in this process. We also note that differences in the mechanical interactions between the cells, the collagen, and the proteoglycans are probably important in the early culture phase, where the pellet is relatively small and the cells are still reasonably closely packed together. At present, however, we believe that the absence of experimental data renders these extensions beyond the scope of feasibility.

Our model was intended to help provide an understanding of the important mechanisms involved in the matrix deposition within pellets. We view this model, which explains favourably the later behaviour, as a first attempt that could be used as a building block for future more complex models. Such models could include interactions of the ECM components, changes in the rate of synthesis due to mechanical structure or stress, and the autocrine induction of additional matrix synthesis as a consequence of a cells’ perceived micro niche. In particular, we believe that the collection of experimental measurement for the concentrations of oxygen or glucose, and for the cell distributions within pellets of different initial sizes would be very useful, in view of possible model extensions with implications therein for further experimental development and regeneration strategies.

## Supporting Information

S1 Text S1 Text contains details of model nondimensionalization. (PDF)
