# Supplementary material for: Extracellular Matrix Deposition in Engineered Micromass Cartilage Pellet Cultures: Measurements and Modelling
Source: PLoS One. 2016 Feb 18;11(2):e0147302. doi: 10.1371/journal.pone.0147302 (PMC4758662; doi:10.1371/journal.pone.0147302)
Supplement: S1 Text — (PDF) [file pone.0147302.s001.pdf]

Extracellular Matrix Deposition in Engineered Micromass Cartilage Pellet Cultures: Measurements and Modelling

Miranda C. Lewis[1], Ben D. MacArthur[1,2], Rahul S. Tare[2], Richard O.C. Oreffo[2], Colin P. Please[3]

[1] Mathematical Sciences, University of Southampton, Southampton, SO17 1BJ, UK

[2] Centre for Human Development, Stem Cells and Regeneration, Faculty of Medicine, University of Southampton, Southampton, SO16 6YD, UK

[3] Mathematical Institute, University of Oxford, Oxford, OX2 6GG, UK

Supporting Information

The mathematical model is given by

$$n + p = 1 \tag{4}$$

$$\frac{\partial B}{\partial t} + \nabla \cdot (\underline{u}\, B) = D\nabla^2 B - \frac{\alpha B}{\kappa_0 + B} H(B)\, n, \tag{5}$$

$$\frac{\partial n}{\partial t} + \nabla \cdot (\underline{u}\, n) = 0, \tag{6}$$

$$\frac{\partial p}{\partial t} + \nabla \cdot (\underline{u}\, p) = \frac{\beta B}{\kappa_0 + B} H(B)\, n \tag{7}$$

with $B = B_0$ and $v = \dfrac{dS}{dt}$ at $r = S$, $v = 0$ and $\dfrac{\partial B}{\partial r} = 0$ at $r = 0$, and $S = S_0$, $n = 1$ and $p = 0$ at $t = 0$.

To nondimensionalise the model, the nutrient concentration is rescaled with that in the culture medium; time with the matrix production rate constant, β; and distance with the nutrient diffusion length scale. The following change of variables is subsequently introduced:

$$B = B_0 \tilde{B}, \quad t = T\tilde{t}, \quad r = L\tilde{r}, \quad S = L\tilde{S} \quad \text{and} \quad v = \frac{L}{T}\tilde{v}$$

where

$$L = \sqrt{\frac{DB_0}{\alpha}} \quad \text{and} \quad T = \frac{1}{\beta}$$

This system can then be simplified by adding together equations (6) and (7), to give the nondimensional model presented in the main text.
